# Correction to ‘Endothelial cell‐derived extracellular vesicles alter vascular smooth muscle cell phenotype through high‐mobility group box proteins’

**DOI:** 10.1002/jev2.70326

**Published:** 2026-06-17

Boyer, M.J., Y. Kimura, T. Akiyama, et al. 2020. “Endothelial cell‐derived extracellular vesicles alter vascular smooth muscle cell phenotype through high‐mobility group box proteins.” *Journal of Extracellular Vesicles* 9: 1781427. https://doi.org/10.1080/20013078.2020.1781427

In the originally published article, the right panel bar graph of Figure 3a had a missing data point (n number should be 5 points in dEV (EV‐depleted serum conditions) instead of 4.The correct bar graph for Figure 3a is as follows:


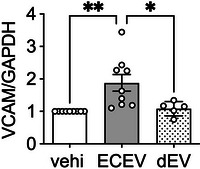


In addition, the section of the Figure legend describing the n numbers should be:

**Figure 3**. Pro‐inflammatory effects of EC EVs on VSMCs. (a and b) Serum starved (48 h) rat aortic VSMCs were incubated with vehicle (vehi: PBS), EC EVs (5 × 10^8^ particles) from either serum‐free conditions or EV‐depleted serum conditions (a) or serum‐free fibroblast. EVs (5 × 10^8^ particles, b) for 6 hours and expression of VCAM‐1 and GAPDH was determined by Western blotting (*n* = 5–9).

The online version of this article has been corrected.

We apologize for this error.

